# The influence of MRAS gene variants on ischemic stroke and serum lipid levels in Chinese Han population

**DOI:** 10.1097/MD.0000000000018065

**Published:** 2019-11-27

**Authors:** Yan Song, Rui Ma, Hongjuan Zhang

**Affiliations:** a Department of Radiology; b Department of Hemodialysis, Jieshou City People's Hospital, Fuyang, China.

**Keywords:** genetics, lipids, MRAS, stroke

## Abstract

Previous studies have indicated that muscle RAS oncogene homolog (MRAS) gene played an important role in cardiovascular diseases. However, the effect of MRAS genetic variations on ischemic stroke (IS) is still not clear. The aim of the current study was to investigate the association between the MRAS polymorphism and IS risk in Han populations.

Three SNPs (rs40593, rs751357, rs6782181) at *MRAS* were selected for genotyping in a sample of 240 IS patients and 430 controls. Logistic regression was performed to evaluate the association of 3 SNPs with IS and IS subgroups.

No association of *MRAS* SNPs with IS risk was observed, while G allele of rs40593 was associated with increased risk of cerebral infarction area. Compared with carriers of the AA genotype, the risk of carriers of the AG+GG genotype increased, with an OR (95%CI) of 2.337 (1.175–4.647), *P* = .016. In relation to lipid profile, rs40593, rs751357, rs6782181 were associated with increased total cholesterol (TC) levels.

Summarily, this study suggested that *MRAS* rs40593 may contribute to the increased risk of area of cerebral infarction of IS in Han population. rs40593, rs751357, and rs6782181 were associated with higher serum TC levels.

## Abstract 2

Supplemental Digital Content is available in the text

## 1 Introduction

Stroke is a major public health disease that has largely contributed to the global burden of disease.^[1]^ According to the Global burden of disease study 2017, the number of stroke patients has grown rapidly from 83 million in 2016 to more than 100 million in 2017, becoming a major disease threatening human health.^[2]^ The available dates suggest that the prevalence of stroke is severe. Stroke is a result of highly complex interaction between lifestyle and genetic factors. Previous studies have confirmed conventional risk factors for stroke, including hypertension, diabetes mellitus, hyperlipidemia, obesity, tobacco use, as well as alcohol drinking.^[3–7]^ In recent years, several genome-wide association studies (GWAS) studies in European populations have successfully identified a new cardiovascular disease risk gene, the muscle RAS oncogene homolog (*MRAS*).^[8,9]^

The MRAS gene is located on the 3q22.3 chromosome and encodes a member of the ras super-family of GTP-binding proteins, which acts on multiple processes of signal transduction, including cell growth and differentiation.^[10,11]^ It is widely distributed in all tissues, especially in the cardiovascular system.^[12]^ Studies have shown that the protein encoded by the MRAS plays an important role in the tumor necrosis factor-alpha (TNF-a) and MAP kinase adhesion signaling pathways, while vascular adhesion molecules involves in atherosclerotic disease by mediating the cellular and intercellular adhesion mechanisms.^[13,14]^ This evidence indicates a potentially pivotal role of *MRAS* in cardiovascular function.

Alshahid et al reported that the *MRAS* rs6782181SNP was associated with increased risk of coronary artery disease (CAD), obesity, hypercholesterolemia, hyperlipidemia and low high density cholesterol (HDL-C) levels in the Saudi population.^[11]^ In the Han population, rs6782181was also found to be associated with elevated serum levels of total cholesterol (TC), triglyceride (TG) and low density lipoprotein-cholesterol (LDL-C).^[15]^ In contrast, another study of Chinese data suggested that the *MRAS* loci might have a minor effect in conferring susceptibility to CAD.^[16]^ It is well recognized that hyperlipidemia and high LDL-C levels are risk factors for stroke^[17]^ and increased stroke mortality.^[18]^ However, the report on *MRAS* and stroke was rare. Therefore, it is necessary to investigate genetic effect of the *MRAS* SNP on stroke susceptibility.

Stroke can be divided into 2 primary categories: ischemic stroke (IS) and hemorrhagic stroke (HS), of which approximately 79% are IS patients.^[2,19]^ In the present study we evaluate the associations of 3 tagging polymorphisms at MRAS with IS risk in Chinese Han population.

## 2 Materials and methods

### 2.1 Study population

We conducted a case-control study involving 240 IS patients with an age >18 years and 430 age group-(3 years) matched subjects free of stroke. The IS cases were consecutively selected from patients admitting to the People's Hospital of Jieshou City in Fuyang (Anhui, China) with a diagnosis of stroke from March to September 2017. The control group was resided in the same communities where the cases were selected from, and were determined to be free of stroke and peripheral atherosclerotic arterial disease based on their medical history, clinical examinations, and electrocardiography.

Approval for this study was granted by the ethics committee of People's Hospital of Jieshou City. Written informed consents were obtained from all subjects or their caregivers.

### 2.2 Diagnosis of stroke and stroke subtype classification

All first-episode stroke cases were diagnosed in accordance with the World Health Organization criteria^[20]^ and confirmed using brain computed tomography (CT) or magnetic resonance imaging (MRI). IS subtypes were determined by Adama criteria system with MRI/CT evidence including large infarction, small infarction and lacunar infarction.^[19]^ Large infarction was defined as cerebral infarction area >30 mm^2^, and involving more than 2 brain anatomical parts of the large blood vessel main blood supply area; small infarction was defined as cerebral infarction area between (15 and 30]mm^2^, and involving 1 small vascular branch occlusion in an anatomical site; lacunar infarction was defined as a lacunar lesion measuring ≤15 mm^2^.

### 2.3 Data collection

The clinical information including the age, sex, smoking and drinking status, body mass index (BMI; weight (kg)/height (m)^2^), medical history and blood pressure (BP) were collected from the subjects’ medical records. Hypertension was defined as a systolic blood pressure of higher than 140 mmHg, and/or a diastolic blood pressure of higher than 90 mmHg, or use of antihypertensive prescription. Hypercholesterolemia was defined as serum total cholesterol >5.2 mmol/L or treatment with a lipid-lowering drugs. Smoking was defined as at least 20 cigarettes per week for 3 months per year. Drinking was defined as at least 2 times per week for 6 months per year.

Peripheral venous blood samples were drawn from subjects after 10 hours of fasting and samples were collected into EDTA tubes. Measurements of TC, LDL-C, HDL-C, TG and glucose (GLU) were performed using commercial kits from BIOSINO (Shanghai, China).

### 2.4 SNP genotyping

The DNA was isolated from peripheral blood leukocytes by a standard protein precipitator method. DNA concentration and purity of each sample were measured using the Thermo Scientific NanoDrop 2000 spectrophotometer. Three MRAS tagSNPs (rs40593, rs751357, rs6782181) were genotyped by using TaqMan-based allelic discrimination assay on the platform of ABI 7900 polymerase chain reaction (PCR) system (Applied Biosystems, Foster City, CA). The nucleotide sequences of primers and fluorogenic probes were presented in supplement Table 1.

### 2.5 Statistical analysis

Quantitative and Categorical variables differences between cases and controls were evaluated by unpaired Student's *t* test and χ^2^ tests, respectively. Hardy-Weinberg equilibrium (HWE) in control group was identified by χ^2^ tests. One-way ANVOA was used to assess the serum lipid levels among SNP different genotypes. The associations between signal SNP and stroke in case-control study were determined by binary logistic regression analysis, and the odds ratio (*OR*) and 95% confidence interval (*CI*) were calculated. Multivariate logistic regression analysis was used to compare the difference between SNP and IS subtypes risk. A *P* value <.05 was considered statistically significant. The statistical analysis was performed with SPSS18.0 (Chicago, IL).

## 3 Results

### 3.1 Demographic and clinical characteristics of participants

The demographic and clinical characteristics of subjects were all presented in Table 1. Of the 240 IS patients, 119 were large infarction, 71 were small infarction and 50 were lacunar infarction. The control group was younger (the mean age of controls was 61.48 ± 9.64 years) compared with IS cases (63.04 ± 9.1 years). As expected, the traditional stroke risk factors such as hypertension frequency, hypercholesterolemia, TG, LDL-C and BMI levels in cases were significantly higher than controls while HDL-C levels was lower in cases (*P* < .05).

**Table 1:**
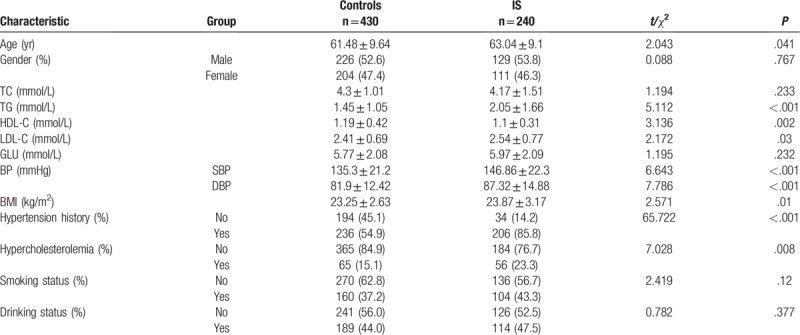
Demographic and clinical characteristics of the case-control study of stroke.

### 3.2 Association analyses of the case-control study for *MRAS* SNP and IS

In this study, the genotype distributions of 3 SNPs were in accordance with HWE (*P* > .05) in the control population. Results of logistic regression analysis showed no association between the *MRAS* SNPs and IS (all *P* > .05, Table 2). Additionally, we also conducted the genetic analyses in each IS subgroups. The G allele of rs40593 was observed to be associated with the increased area of cerebral infarction. Compared with carriers of the AA genotype, risk of carriers of the AG+GG genotype increased [(OR (95%CI): 2.337 (1.175–4.647), *P* = .016)]. After adjustment of age, gender, TC, TG, HDL-C, LDL-C, GLU, BMI, drinking and smoking status, the association was still significant (*P* = .032, Table 3).

**Table 2:**
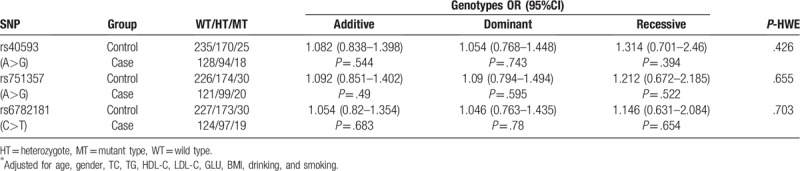
Association analyses of *MRAS* and IS.

**Table 3:**
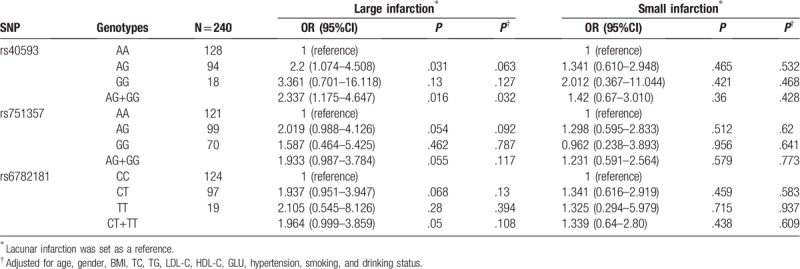
Association between *MRAS* and IS subgroups.

### 3.3 Correlation analysis of *MRAS* SNP and serum lipid levels

**Figure 1:**
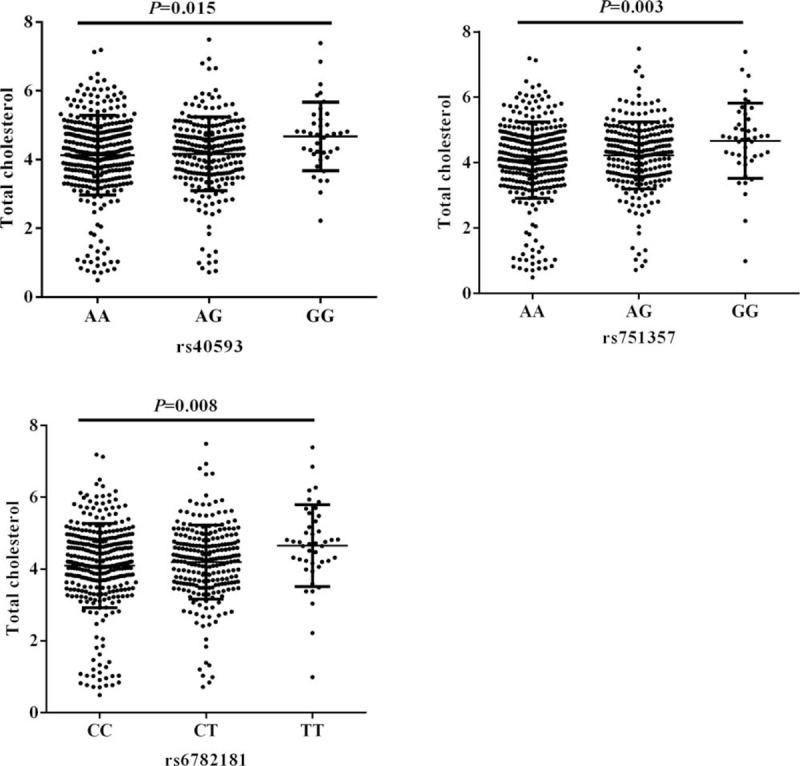
Serum total cholesterol (TC) levels among rs40593, rs751357, and rs6782181 genotypes in the 621 subjects with taking no lipid-lowering drugs. TC levels were plotted around the median as box plots, where the dots represented individual data. Diamonds and the whiskers represented the mean and SD of TC levels. Figure (A), (B), and (C) presented correlations among rs40593, rs751357, and rs6782181 genotypes, respectively.

We further assessed the TC, TG, LDL-C and HDL-C levels among the SNPs genotypes. After excluding the population who were taking lipid-lowering drugs, 621 people were analyzed finally. Variants of rs40593, rs751357, and rs6782181 were associated with TC levels, but no differences were observed with TG, LDL-C and HDL-C levels (Supplement Table 2). For the 3 SNPs, carriers of the minor allele genotypes showed higher TC levels, *P* was .015, .003, and .008, respectively (Fig. 1).

## 4 Discussion

The associations between *MRAS* polymorphisms and cardiovascular diseases have been a matter of interest in recent years. A GWAS research of European populations has revealed a new susceptibility locus for CAD in the region of *MRAS* gene, rs9818870.^[8]^ Similar finding was observed by Mehta et al.^[21]^ Some researchers have suggested that impairment of endothelial function might be a relevant cause for the reported association of rs9818870 with CAD risk; however, this explanation failed to be confirmed.^[22]^ More recently, Alshahid et al has reported that another *MRAS* SNP (rs6782181) was associated with an increased risk of CAD in the Saudi populations.^[11]^ An inconsistent result that the *MRAS* loci might have a minor effect in conferring susceptibility to CAD was also observed in a Chinese study.^[16]^ Hubacek et al demonstrated that the rs9818870 variant was not associated with acute coronary syndrome or mortality in the Czech Slavonic populations.^[23]^ Despite the plenitude of descriptive data on genetic predisposition to CAD, the association study of *MRAS* and stroke was limited.

In the current study, we assessed the relationship between 3 variants (rs40593, rs751357 and rs6782181) at *MRAS* and IS risk. No association was found between *MRAS* and IS, while the G allele of rs40593 was observed to be associated with the increased area of cerebral infarction in IS group. After adjustment of age, gender, TC, TG, HDL-C, LDL-C, GLU, BMI, drinking and smoking status, the association was still significant. SNP rs40593 is localized in the 3’-UTR of *MRAS* close to a cluster of regulatory miRNA binding sites, which is increasingly considered to regulate the *MRAS* expression, translation and MRAS protein levels. It is well known that MRAS has been shown to be involved in adhesion signaling, which indicates an important relevance in the atherosclerotic process.^[24]^ The mechanism seems to be that rs40593 combines with miRNA, leading to changes in the level of MRAS.

Additionally, in order to better understand the biological characteristics of these loci, we studied whether the SNPs are related to traditional stroke risk factors, or associated with other human disease traits. To the best of our knowledge, the association between the *MARS* and serum lipid levels is little known. The variant rs6782181GG genotype has been associated with the risk of hypercholesterolemia, hypertriglyceridemia and low HDL-C levels.^[11]^ In the Han population, rs6782181 was found to be associated with elevated serum levels of TC, TG, LDL-C in males, and higher serum TC and LDL-C levels in Mulao populations.^[15]^ Our work partly confirmed the results that carriers of rs6782181 variant had higher TC levels. Furthermore, rs40593 and rs751357 variant presented positive correlation with TC levels in current study. It is well recognized that TC level elevation is a major health problem associated with an increased risk of cardiovascular diseases.^[25,26]^ These findings provided a potential mechanism for the association between *MRAS* and cardiovascular diseases.

Several limitations need to be considered. Firstly, none of the SNPs showed a statistically significant association with IS risk in this study, and relatively small sample size may be responsible for the lack of association. Secondly, we were incapable of measuring the MRAS protein levels which made us could not deeply investigate the relationship between SNPs mutation and protein levels. Thirdly, no biological function of *MRAS* variants was investigated.

In summary, this study provides an evidence that *MRAS* rs40593 variant may contribute to the risk of increased area of cerebral infarction of IS in Han population. Variants of rs40593, rs751357, and rs6782181 were associated with higher serum TC levels. Further independent studies with large sample size are needed to confirm our findings.

## Author contributions

**Conceptualization:** Hongjuan Zhang.

**Data curation:** Yan Song, Rui Ma.

**Formal analysis:** Rui Ma.

**Project administration:** Hongjuan Zhang.

**Writing – original draft:** Yan Song.

**Writing – review & editing:** Hongjuan Zhang.

## Supplementary Material

Supplemental Digital Content

## Supplementary Material

Supplemental Digital Content
