# Supplementary material for: The influence of MRAS gene variants on ischemic stroke and serum lipid levels in Chinese Han population
Source: Medicine (Baltimore). 2019 Nov 27;98(48):e18065. doi: 10.1097/MD.0000000000018065 (PMC6890362; doi:10.1097/MD.0000000000018065)
Supplement: Supplemental Digital Content [file medi-98-e18065-s001.docx]

| Supplement table1Nucleotide sequences of primers and fluorogenic probes | | |
| --- | --- | --- |
| SNPs | Probe sequence | Primer Sequence |
| rs40593 | FAM-ATCACCAGCACTGAG-MGB | 5’TTCTGAAAATGTGCTGTGTTCCTAC3’ |
|  | HEX-ATCACCAGCGCTGAG-MGB | 5’TGATTTCCAACAAGCAAAAGTCTCA3’ |
| rs751357 | FAM-AGTCCTCCTTCCTC-MGB | 5’GGTTCACCACTCCAAAGCTAGGT3’ |
|  | HEX-CCAGTCCTTCTTCCT-MGB | 5’TGCAAAGGTCTGTGAAGCAGAT3’ |
| rs6782181 | FAM-ATGAATATCTTCAGGAATG-MGB | 5’AGAGTGAGTGCTCATGGGTAAGG3’ |
|  | HEX-ATGAATACGTTCAGGAAT-MGB | 5’TCAGTGGTTTTTAGTGTATTCACAGAGTT3’ |
