# Supplementary material for: The influence of MRAS gene variants on ischemic stroke and serum lipid levels in Chinese Han population
Source: Medicine (Baltimore). 2019 Nov 27;98(48):e18065. doi: 10.1097/MD.0000000000018065 (PMC6890362; doi:10.1097/MD.0000000000018065)
Supplement: Supplemental Digital Content [file medi-98-e18065-s002.docx]

| Supplement table 2 lipid levels (mmol/L)among different genotypes | | | | | | |
| --- | --- | --- | --- | --- | --- | --- |
| SNP | Genotypes | N=621 | TC | TG | LDL-C | HDL-C |
| rs40593 | AA | 347 | 4.13±1.15 | 1.66±1.37 | 2.4±0.7 | 1.14±0.39 |
|  | AG | 235 | 4.16±1.07 | 1.52±1.13 | 2.4±0.64 | 1.17±0.38 |
|  | GG | 39 | 4.67±1.00 | 1.72±1.36 | 2.61±0.83 | 1.13±0.34 |
|  | F |  | 4.239 | 1.049 | 1.596 | 0.523 |
|  | *P* |  | 0.015 | 0.351 | 0.203 | 0.593 |
| rs751357 | AA | 332 | 4.1±1.17 | 1.66±1.36 | 2.38±0.7 | 1.14±0.39 |
|  | AG | 245 | 4.2±1.03 | 1.51±1.13 | 2.42±0.65 | 1.16±0.38 |
|  | GG | 44 | 4.65±1.14 | 1.8±1.4 | 2.63±0.81 | 1.15±0.34 |
|  | F |  | 5.895 | 1.327 | 2.797 | 0.349 |
|  | *P* |  | 0.003 | 0.266 | 0.062 | 0.705 |
| rs6782181 | CC | 336 | 4.08±1.17 | 1.65±1.36 | 2.37±0.7 | 1.14±0.39 |
|  | CT | 241 | 4.22±1.02 | 1.52±1.14 | 2.44±0.65 | 1.16±0.38 |
|  | TT | 44 | 4.67±1.15 | 1.8±1.4 | 2.62±0.79 | 1.17±0.38 |
|  | F |  | 4.9 | 1.352 | 2.481 | 0.085 |
|  | *P* |  | 0.008 | 0.26 | 0.085 | 0.919 |
